# On the Formation of Formic Acid from Carbonic Acid Gas

**Published:** 1856-06

**Authors:** 


### *On the Formation of Formic Acid from Carbonic Acid Gas.*

By Berthelot.

(Translated for the Examiner.)

Carbonic acid gas stands, in certain respects, in the same relation to formic acid that olefiant gas does to alcohol,

$$C_2\,O_2 = C_2\,H_2\,O_4 - 2HO\,;\; C_4\,H_4 = C_4\,H_6\,O_2 - 2HO$$

By heating with concentrated sulphuric acid, alcohol affords olefiant gas ; formic acid, carbonic oxide gas. Guided by these considerations, Berthelot attempted to produce formic acid from carbonic oxide, in the same manner as he had done in the regeneration of alcohol by means of sulphuric acid (Annalen, xciv.

78). The formation of formic acid from carbonic oxide did, in fact, take place when the latter body was allowed to act at an elevated temperature upon potassa. Ten gram. of slightly moistened potassa were put in a globe of ½ litre contents; the globe then filled with carbonic oxide gas, the opening closed by melting over the lamp and heated for 40 hours in a water bath. When opened under quicksilver, the latter fills the globe completely, the carbonic oxide gas has been absorbed, and if the contents of the globe be now dissolved in water, the solution saturated with sulphuric acid and distilled, dilute formic acid passes over.—*Annalen der Chemie,* Jan., 1856, *from Compt. Rendus.*

